# Supplementary material for: Tunable narrowband excitonic Optical Tamm states enabled by a metal-free all-organic structure
Source: Nanophotonics. 2022 Nov 7;11(21):4879–88. doi: 10.1515/nanoph-2022-0419 (PMC11502094; doi:10.1515/nanoph-2022-0419)
Supplement: Supplementary file 1 — Supplementary Material Details [file j_nanoph-2022-0419_suppl_001.docx]

**Tunable narrowband Excitonic Optical Tamm States enabled by a fully organic structure.**

*Miguel Castillo, Diogo Cunha, Carla Estevez-Varela, Daniel Miranda, Isabel Pastoriza-Santos, Sara Nuñez-Sanchez*, Mikhail Vasilevskiy, Martin Lopez-Garcia**


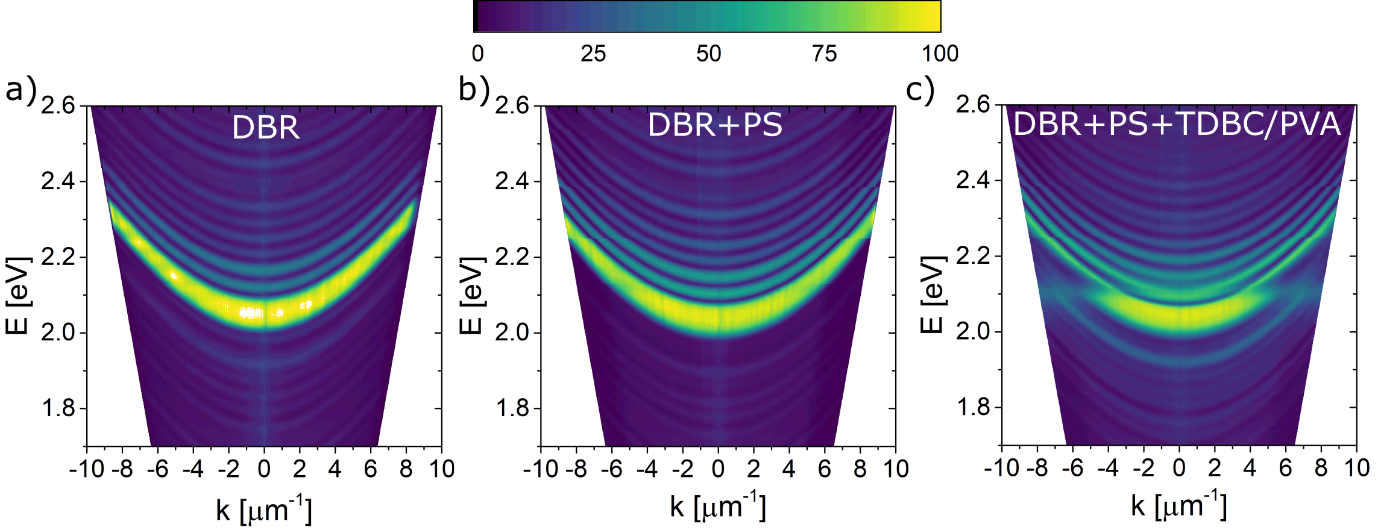


**Figure S1: p polarised reflection measurements**. Angle-dependent reflection spectrum (p polarised) of the DBR (a), DBR with thin PS layer (b) and DBR with thin PS layer and TDBC-PVA (c), represented in figure 1b, with *Δ=196* nm of the main manuscript.


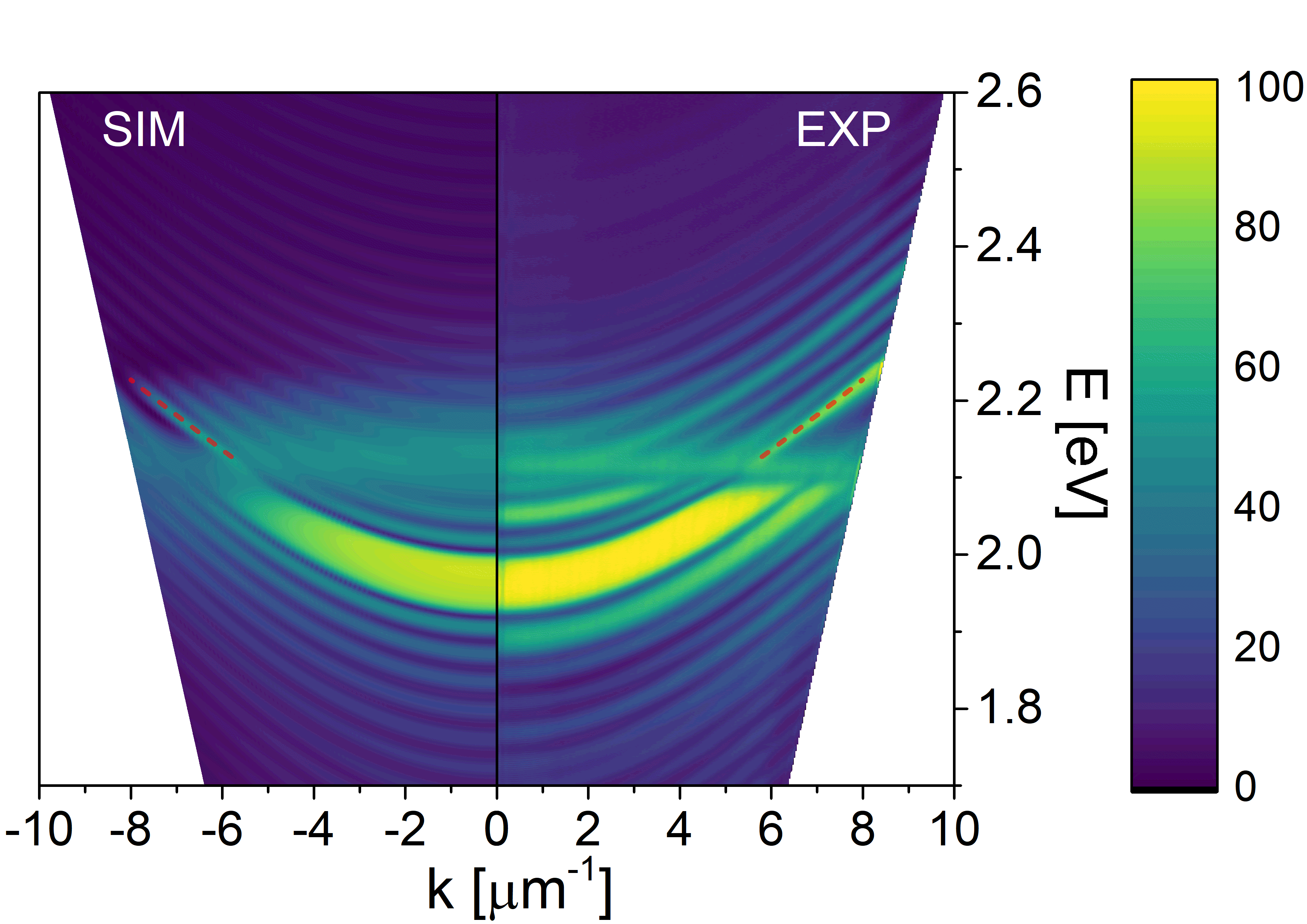


**Figure S2: Reflection of *204* nm period sample.** Simulated and measured angle-resolved reflection spectrum (s polarised) of the *Δ=204* nm DBR sample. Red dashed lines represent the solution from equation S8.


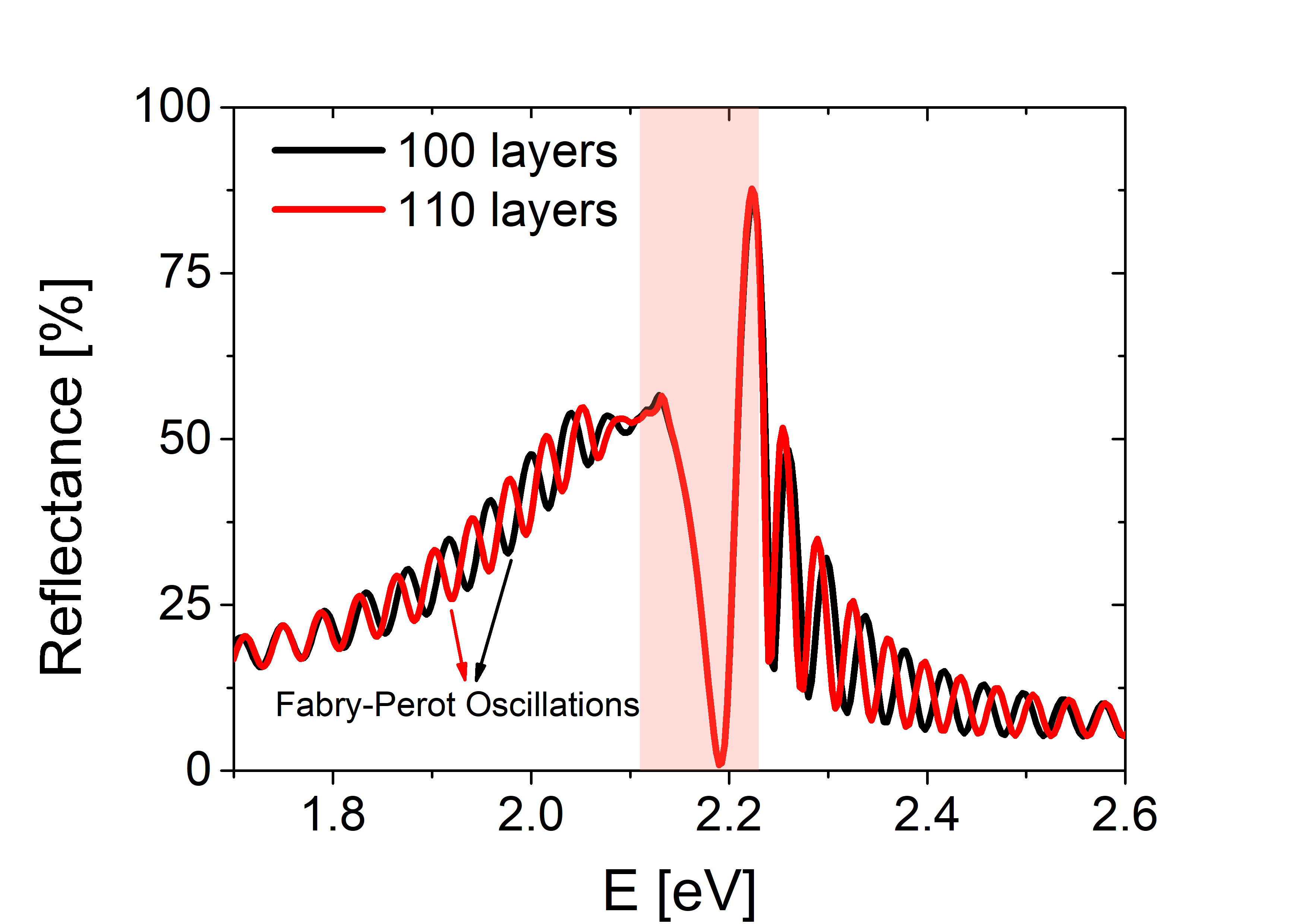


**Fig S3. Fabry-Perot oscillations.** S polarised reflection at *35*° of two DBRs with *Δ=196* nm with a different number of layers. The shaded region represents the optically metallic-like area of TDBC-PVA where a Tamm state can occur. The Fabry-Perot oscillations do not match as a consequence of the different finite sizes of the structures.


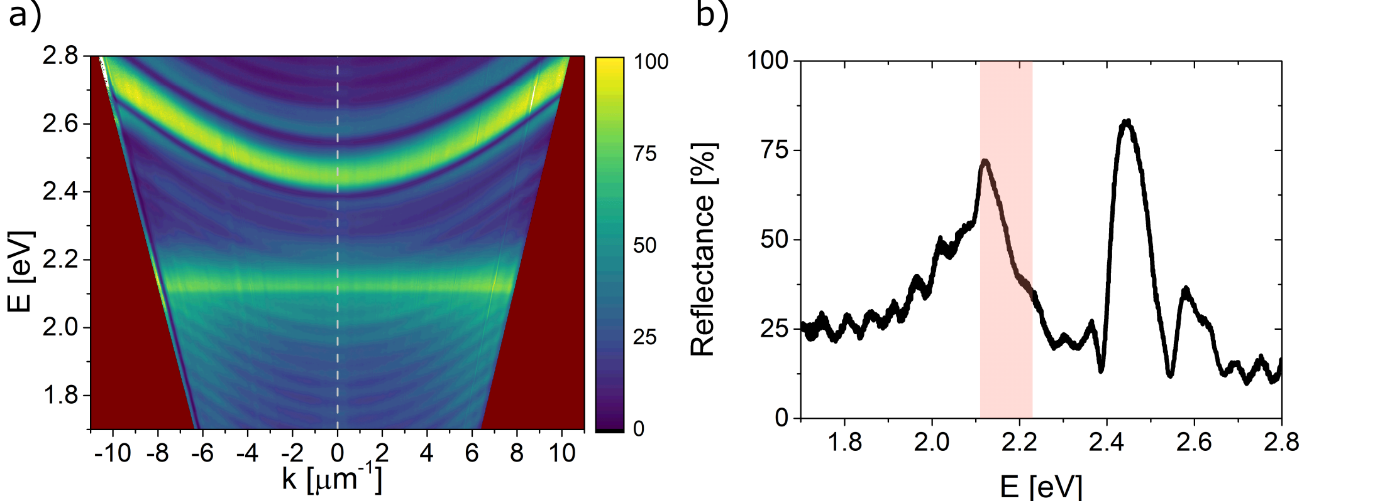


**Fig S4. Detuned photonic bandgap.** Angle dependent (s polarised) (a) and normal incidence (b) reflection spectrum of a structure similar to that represented in figure 1 of the main manuscript, but with a detuned photonic bandgap and metal-like optical properties of TDBC-PVA. This detuned DBR has thicknesses *d_PVA_=83* nm and d_PS_=*78* nm (producing a photonic bandgap at *2.48* eV at normal incidence).


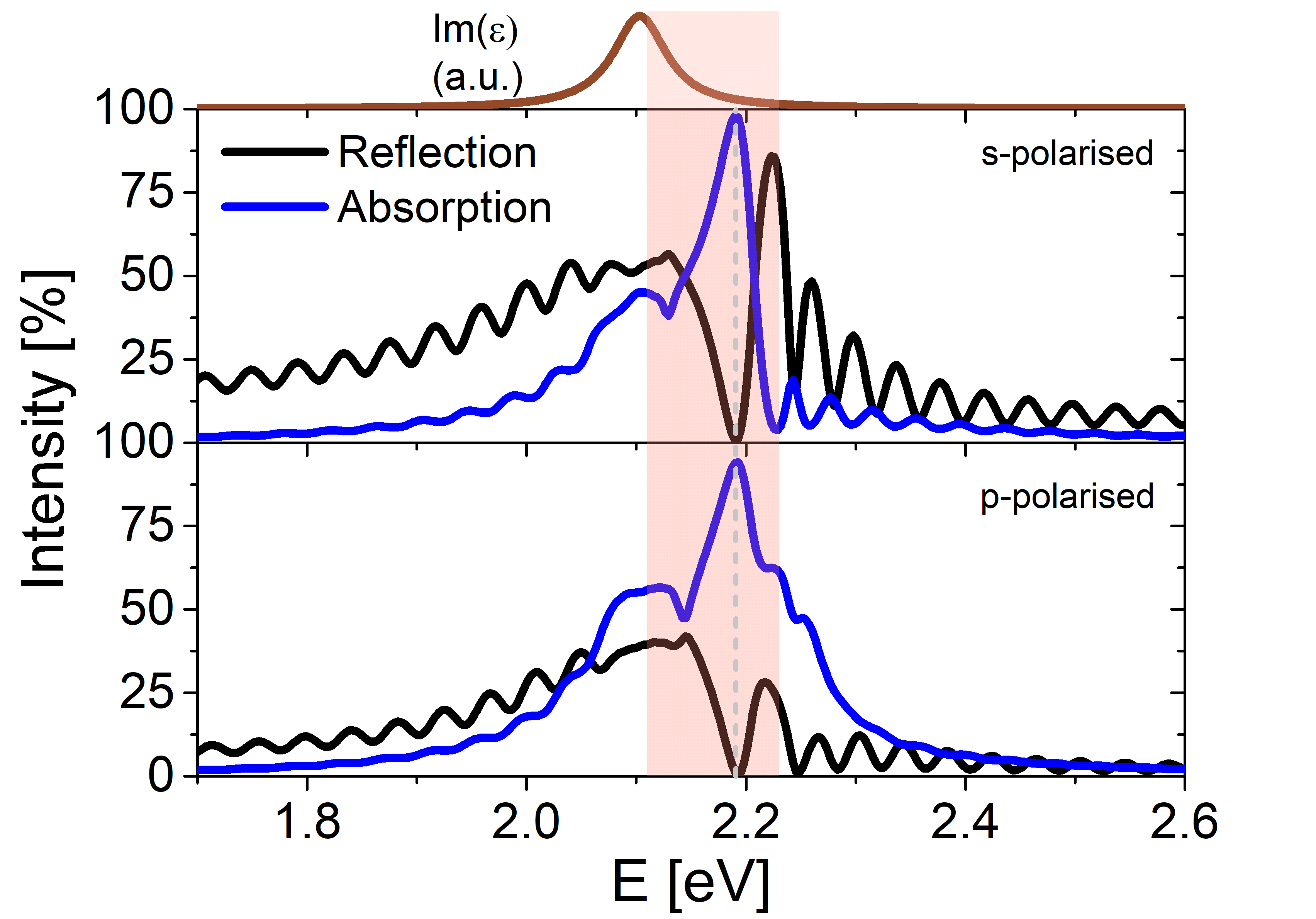


**Figure S5. Tamm state enhancing absorption.** Simulated s- and p-polarised reflection and absorption at $\theta=$ *35*° of the structure in figure 1 with *Δ=196* nm. The shaded region represents the optically metallic-like area of TDBC-PVA and the vertical grey dashed line represents the minimum in reflection.


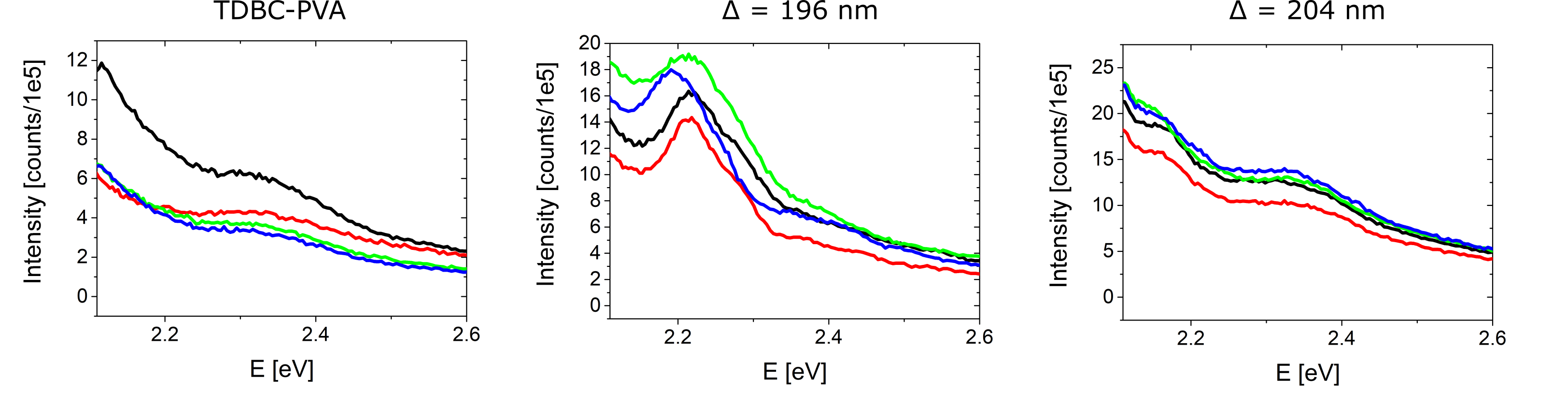


**Figure S6. PLE of samples.** Raw data of the PLE measurements of a single TDBC-PVA layer and of the OTS structures. Each line corresponds to a different measurement on a different spot of the sample. The results shown in figure 3b are the result of an average of these measurements normalised to be 1 at the exciton energy.

**Theoretical calculation of the Optical Tamm state**

To calculate the reflectance of these structures, we make use of the transfer matrix formalism. It allows us to evaluate the Fresnel coefficients from the matrix *T_ij_* that transforms in-plane components of the incident electromagnetic field into the transmitted ones. For the *s*-polarization, the Fresnel coefficients are written as:

$$\hat{t}_{s}=\frac{2}{T_{11}^{-1}+\frac{ck_{3x}}{\omega}T_{12}^{-1}+\frac{\omega}{ck_{1x}}T_{21}^{-1}+\frac{k_{3x}}{k_{1x}}T_{22}^{-1}} (S1)$$

$$\hat{r}_{s}=\left( T_{11}^{-1}+\frac{ck_{3x}}{\omega}T_{12}^{-1} \right)\hat{t}_{1}-1 (S2)$$

where *k_1(3)x_* is the wave vector of the incident (transmitted) wave and $T_{ij}^{-1}$ are the matrix elements of the inverse transfer matrix of the whole structure,

$$T^{-1}=T_{TDBC}^{-1}.T_{DBR}^{-1}, (S4)$$

with $T_{DBR}^{-1}=\left( T_{PVA}^{-1}.T_{Ps}^{-1} \right)^{N}$, where $N$ is the number of pairs of layers (i.e. periods) constituting the Bragg reflector and the individual matrices, for a medium A, are written as

$$\hat{T}_{A}=\left( \begin{matrix} \cos\left( k_{Ax}d_{A} \right) & i\frac{\omega}{ck_{Ax}}\sin\left( k_{Ax}d_{A} \right) \\ i\frac{ck_{Ax}}{\omega}\sin\left( k_{Ax}d_{A} \right) & \cos\left( k_{Ax}d_{A} \right) \end{matrix} \right). (S5)$$

Here *d_A_* is the thickness of the layer and $k_{Ax}={(\varepsilon_{A}{(\omega/c)}^{2} -k^{2})}^{1/2}$ with *k* being the transversal wave-vector and c the light velocity.

With the TDBC-PVA layer included in the structure, a minimum in the reflectance spectra appears within the optical metal-like region of TDBC-PVA, which is a fingerprint of the Tamm state. The OTS is an eigenstate of the whole structure and it can be shown that its frequency, for a given $k$, can be found by equating the denominator of Eq. (S1) to zero.^[37]^ This condition can be cast in the following form:^[33]^

$$\hat{r}_{1}\hat{r}_{2}e^{2ik\delta}=\left| \hat{r}_{1} \right|e^{i\varphi_{1}}\left| \hat{r}_{2} \right|e^{i\varphi_{2}}e^{2ik\delta}=1 (S5)$$

where $\hat{r}_{1}$ ($\hat{r}_{2}$) corresponds to the Fresnel reflection coefficient of the heterostructure 1(2) alone (in vacuum) and *δ* is the thickness of the buffer layer. For simplicity, we will consider that the bottom mirror is the heterostructure 2 (dictated by the reflection coefficient $\hat{r}_{2}$) and the heterostructure 1 is the TDBC-PVA layer alone. It means that, under these assumptions, we can consider the limit of $\delta\to$*0* and Eq. (S5) reduces to:

$$\hat{r}_{1}\hat{r}_{2}=\left| \hat{r}_{1} \right|e^{i\varphi_{1}}\left| \hat{r}_{2} \right|e^{i\varphi_{2}}=1 (S6)$$

From Eq. (S6) we have:

$$\left\{ \begin{aligned} \left| \hat{r}_{1} \right|\left| \hat{r}_{2} \right|=1 (S7) \\ \varphi_{1}+\varphi_{2}=2\pi m (S8) \end{aligned} \right.$$

where *m* is an integer and *φ_(1(2))_* is the argument of the complex number $\hat{r}_{1(2)}$. Eq. (S8) is known as the phase-matching condition and it determines an implicit dispersion relation of the OTS. For semi-infinite DBR and TDBC-PVA, with the imaginary parts of the permittivity neglected, Eq. (S7) is obeyed automatically and the phase-matching condition (S8) yields a real eigen-frequency. To calculate it, we need to take the limit *N→∞* for evaluating the DBR transfer matrix (which is facilitated by using the Chebyshev formula) and, for the TDBC-PVA layer, take the reflection Fresnel coefficient as

$$\hat{r}_{1}=\frac{k_{1x}-k_{2x}}{k_{1x}+k_{2x}} , (S9)$$

where *k_1x_* and *k_2x_* are the wave-vector’s normal components in air and TDBC-PVA, respectively.
